# Clinical Coding Audit: No coding – No Income – No Hospital

**DOI:** 10.7759/cureus.10664

**Published:** 2020-09-26

**Authors:** Jonathan White, Muhammad Jawad

**Affiliations:** 1 Acute Internal Medicine, East Surrey Hospital/Surrey and Sussex Healthcare National Health Service Trust, Redhill, GBR

**Keywords:** clinical coding, medical coding, economics, funding, financial management, hospital management

## Abstract

Aim: Audit assessing the accuracy of clinical coding by trust guidance on revenue generation.

Method: Clinical coding form containing common clinical codes placed at the front of patient notes between July 2014 and August 2014 on a respiratory ward. Medical staff, preferably registrars or consultants, recorded the primary diagnoses and comorbidities on the form. Completed forms sent to the clinical-coding department who supplied the tariff generated. Revenue generated during the test period was compared with the prior two months.

Results: Comparable discharges between periods with average revenue generated a difference per patient of £339, totalling a £36,273 increase per month.

Conclusion: Simple diagnoses and comorbidities coding forms completed by a senior doctor helped junior doctors improve documentation and accuracy at discharge whilst generating more revenue for the trust.

## Introduction

Clinical coding is the process of recording diagnoses, treatment, investigations, and procedures that occurred during an episode of care for a patient; within primary, secondary, or tertiary care. The standardised and comparable codes are recognised both nationally and internationally to compile an anonymous dataset that can be used in multiple ways for statistical analysis to hospital reimbursement and health services planning. Since April 2017, National Health Service (NHS) Digital [1] states that the standardised codes are taken from International Classification of Diseases 10th revision (ICD-10) and Office of Population Censuses and Surveys Classification of Surgical Operations and Procedures 4th version (OPCS-4) to ensure congruity with international standards. The NHS operates under a Payment by Results system which translates the clinical codes into groupings per trust that correlates to a tariff for payment generation of the hospital for services provided. The fundamental information underpinning this process is the documentation by the clinicians whereby only certain information can legally be coded, for example, diagnoses preceded by “likely”, “possible”, “?” are not allowed to be coded. Hence if this were the discharge diagnosis it could not be used to generate revenue and the trust would not receive remuneration for services provided [2,3]. In addition, there is a timeframe in which information must be coded and if breached then payment cannot be sought for that patient-episode (a single continuous period of attendance at a healthcare provider for a patient). This demonstrates how easily revenue could be lost for the healthcare provider for one patient-episode; extrapolate that out for either the same patient attending multiple times, for a ward of patients, or for a whole time period such as “the winter flu crisis” every year and it is clear how much potential revenue could be lost solely due to incomplete, inaccurate, ambiguous, or illegible documentation, or lack of knowledge of this system by clinicians. For clarity, an example with financial matching to the clinical coding:

Incomplete coding: Gastroenteritis and colitis & Intestinal infectious disorder with a length of stay 2+ days without major co-morbidity (co-morbidity not coded). Tariff = £1,797. Complete coding: Gastroenteritis and colitis & Volume depletion (dehydration) & Intestinal infecious disorder with length of stay 2+ days with major co-morbidity (co-mobidity coded). Tariff = £3,965.

There are further intricacies in the system as not all healthcare providers have the same multiplier attached to the coded tariffs due to institutional overhead costs; for example, a GP practice will not earn the same remuneration for the same codes as a secondary or tertiary care hospital trust.

## Materials and methods

**Figure 1:**
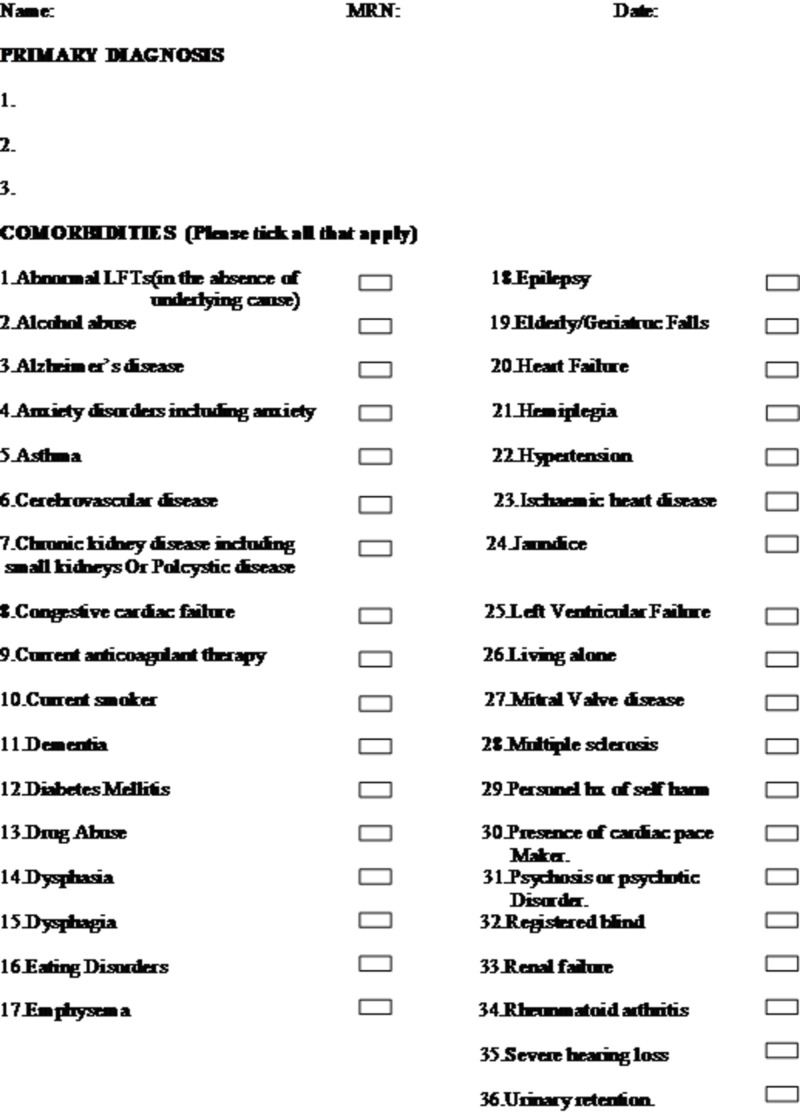
Standardised form for coding including the most common co-morbidities and space for primary diagnoses

A form (Figure 1) was produced with common clinical co-morbidities, mainly those found in patients that are cared for and present requiring respiratory ward treatment, for coding and a section for three primary diagnoses, including patient identifiers. The primary diagnoses were to be completed by a registrar or consultant to ensure accuracy was maintained and that they would be the clinician leading the treatment management plan. The forms were used between July 2014 and November 2014 on the respiratory ward (Tilgate ward) at East Surrey Hospital, Reigate.

During this testing period, these forms were sent, along with the patient notes at discharge, to the clinical coding department which supplied the tariffs generated according to the completed form. The intervention period, July to November 2014, was then analysed and compared to the control period, May to June 2014, in order to derive the financial implications of more accurate complete coding specific for this ward.

## Results

**Figure 2:**
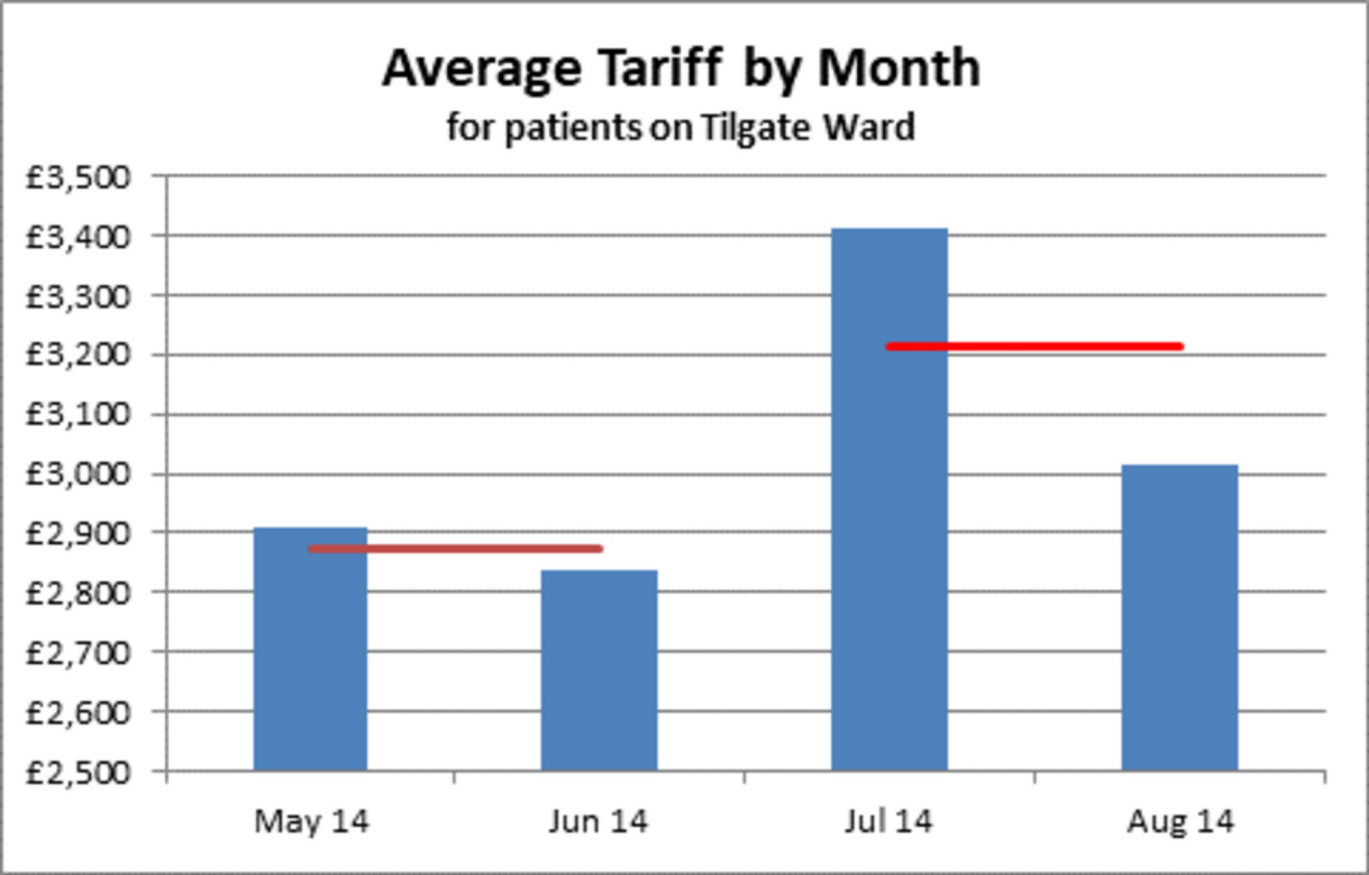
Bar chart showing the sum total per month, from May 2014 to Aug 2014 (blue bars) and the average tariff for each period (red line)

In the control period between May and June 2014, there were 129 and 98 patients discharged, respectively; the average tariff generated per patient for May and June was £2,911 and £2,840, respectively. During the intervention period of July and August 2014, there were 96 and 93 discharges, respectively; and an average tariff generated per patient of £3,411 and £3,014, respectively (Figure 2). By taking the average tariff we were able to minimise the discharge differences of the two periods.

The average during the control period was £2,875, and during the intervention period, from July to August, it was £3,212 (an 11.7% increase). Comparing the two periods, there was an average tariff difference per patient of £339 more during the intervention period. The control period of two months had a total of 227 patients discharged; applying the average tariff per patient financial uplift would generate roughly an extra £38,477 per month for this ward and henceforth the hospital.

## Discussion

The decision to introduce this diagnosis cover sheet resulted from an in-depth knowledge of how healthcare trusts generate finance to be able to continue functioning and cover the continually escalating costs of modern-day healthcare. The main source of income for healthcare trusts come from a payment structure called “Payment by Results” (PbR) which was slowly phased into the healthcare payment structure between 2003 and 2009. The driving factor for this change was to promote more efficient care and help match trust costs with services provided to the individual patient-episode. The previous hospital payment structure was based on historical budgetary data and provided the hospital with a fixed total annual budget; this was not considered to incentivise attracting extra patients nor increasing patient activity levels [3]. The then new PbR system meant that with increasing patient-episodes and increasing resources being used by each hospital trust the overheads could be reclaimed more accurately and help develop better more equitable services for their catchment community demographics [4]. The PbR system has been continually updated in line with current emerging research, evidence, and policy to promote and ensure patient-centred care, as well as safely attempting to reduce the financial strain within the healthcare system by rewarding in-house efficiency and adherence to best practice policies [4].

Nonetheless, unfortunately, it seems that the PbR system, with profit or loss, does not greatly (or at all) influence individual hospital specialties to become more efficient and help reduce overhead costs or to generate more revenue for the hospital. There have been a few trusts nationally that have implemented specialist department budgets and service line reporting using the PbR system. Specialty department costs account for the vast majority of hospital costs, however, this is rarely considered by the clinical team leading to disparity in goal-aims [5]. This can clearly be seen by the difference in revenue generated by introducing a simple diagnosis cover sheet to improve clinical coding as in this article.

The results are very clear on the financial efficacy of introducing this simple diagnosis and co-morbidity covering sheet completed by a senior clinician, showing on average an 11.7% increase on revenue generated over the two-month intervention period, which equated to roughly £38,500 more per month. If we were to extrapolate this over a year, assuming similar discharge numbers and coding tariffs, this would be an increase of almost £500,000. These figures represent the potential income of one ward within our hospital. With a further 16 inpatient wards, and two to three short-stay wards of similar size, the tariff generated per patient in our hospital could represent a revenue increase estimated at £9 million per year.

Limitations and future research

The main limitation of this intervention is that it was conducted over a relatively short period of time and only included a single respiratory ward. A longer duration such as a year would have included episodes like “the winter flu crisis” and the relatively reduced pressures over summer, and would have produced a more complete analysis for comparison. Inclusion of multiple wards, or the whole hospital, would have yielded more powerful data for the potential amplified revenue that had previously been lost and would provide a better budgetary indication of the earning potential of the hospital, as long as coding standards were maintained. There is good scope for this to be tested on multiple different wards to ascertain whether there could be any benefit to implementing this on every ward, including use of this cover sheet at different points in the year, or testing it for a yearlong period.

The intervention did not consider the financial impact of clinical coding conscientious documentation in daily clinical notes. As mentioned in the introduction, there are certain restrictions on what can and cannot be coded for a patients care episode, such as the use of “likely”, “impression”, “possible”, and “?” means the following words/diagnoses must be withheld from the clinical codes. Most clinical documentation is dictated/derived from senior clinicians to the more junior clinicians. If a hospital policy were to be implemented where the above words were banned from notes and instead phrases such as “treat as”/”treat for”/”working diagnosis”, if unsure, were used to signify the direction of treatment this would allow the coding team to input this into the care episode and generate more revenue, given that the coding team is unable to alter wording even if there is clear intent or direction of treatment. Other suggestions to maximise tariffs generated would be: accurate and comprehensive discharge summary; list procedures performed; list all co-morbidities; avoid ambiguous abbreviations (e.g. MS could be multiple sclerosis or mitral stenosis); document the drug from overdoses or any adverse reactions to medications; name the pathogen cultured or treating for; be specific and document the result of any and all abnormal investigations; specify the failing organ and severity of failure; and list the drugs you are prescribing, especially high-cost medications. This list is not exhaustive but could easily further increase clinical coding efficacy and hence care episodes becoming more profitable to the trust.

As mentioned above, most documentation is transcribed from senior clinicians and in the fast-paced and complex world of medicine, these changes could easily be overlooked or deemed non-essential. The authors would advocate that these minor changes in documentation-habit could generate greatly increased revenue for the trust. It could either prevent a trust from huge financial deficit or, being optimistic, potentially generate a more favourable budget or bargaining power for the department enabling it to purchase equipment for better patient care or to increase its resources available to patients. Senior clinicians, as well as juniors, should be educated on the importance of the PbR system used by the NHS to generate hospital revenue. This would ensure that the trust does not potentially haemorrhage money unnecessarily, and that accurate complete documentation will further enable the trust to improve services and resources, not just for patients but also for staff.

## Conclusions

The introduction of a cover sheet for clinical coding listing the diagnoses and most common co-morbidities of each patient going through a respiratory ward over an intervention period of two months raised an additional £38,477 per month (11.7%) compared to a similar control period two months prior. This increased revenue could be further extrapolated to roughly £500,000 more revenue generated over a yearly period. This showed the effectiveness of this simple intervention of this covering sheet completed by a senior clinician. Further to this, there is the payment structure of the NHS, Payment by Results, which informs the money received by trusts to pay for the services it provides. This is poorly known, or unknown, by most clinicians which leads to large amounts of lost revenue, and active engagement and training could help alleviate this issue. Accurate and conscientious clinical coding is extremely important, especially in the current climate whereby trusts are struggling to finance the services they provide, and by knowing a few simple rules as to what can and cannot be coded could generate greatly increased revenue for the trusts which can then be disseminated to the workings of the trust.
